# Long-term response-stimulus associations can influence distractor-response bindings

**DOI:** 10.5709/acp-0158-1

**Published:** 2014-06-26

**Authors:** Birte Moeller, Christian Frings

**Affiliations:** Department of Psychology, University of Trier, Germany

**Keywords:** long-term associations, short-term stimulus- response bindings, distractor-response binding, action control, learning

## Abstract

Strong associations between target stimuli and responses usually facilitate fast and effortless reactions. The present study investigated whether long-term associations between distractor stimuli and responses modulate behavior. In particular, distractor stimuli can affect behavior due to distractor-based stimulus-response retrieval, a phenomenon called *distractor-response binding*: An ignored stimulus becomes temporarily associated with a response and retrieves it at stimulus repetition. In a flanker task, participants ignored left and right pointing arrows and responded to a target letter either with left and right (strongly associated) responses or with upper and lower (weakly associated) responses. Binding effects were modulated in dependence of the long-term association strength between distractors and responses. If the association was strong (arrows pointing left and right with left and right responses), binding effects emerged but only in case of compatible responses. If the long-term association between distractors and responses was weak (arrows pointing left and right with upper and lower responses), binding was weaker and not modulated by compatibility. In contrast, sequential compatibility effects were not modulated by association strength between distractor and response. The results indicate that existing long-term associations between stimuli responses may modulate the impact of an ignored stimulus on action control.

## INTRODUCTION

We are surrounded by many different and constantly changing stimuli in our everyday life. Yet, most of the times we are able to respond to the currently relevant stimulus very quickly and effortlessly. For example, if the traffic light you are approaching turns red, you automatically start slowing down your car - in order to do so, you don’t even have to stop talking to your passengers. Apparently, many objects and stimuli that we encounter repeatedly have been associated with a certain response which can be retrieved automatically (see Logan, 1988, 1990). As a con-sequence, with more automatization, continuously less attention is necessary to respond in an accurate way (e.g., Hasher & Zacks, 1979; Logan, 1979; Shiffrin & Schneider, 1977). Further, it has been proposed that a stimulus can trigger its automatized response even if the stimulus is not attended to (see Kahneman & Treisman, 1984; Kornblum, 1994; Kornblum, Hasbroucq, & Osman, 1990). Interestingly, retrieval theories of action control assume automatic processes of stimulus-response integration and retrieval after only a single encounter of a stimulus-response episode. In contrast to associations due to automatization via repeated pairings, these stimulus-response bindings are relatively short lived. Depending on the specific setting, such *short-term associations* between stimuli and responses hold for about 1 to 6 s (Frings, 2011; Herwig & Waszak, 2012).

An underlying mechanism that has been proposed to (at least partially) account for short-term bindings is the synchronization of firing patterns of neural units that represent features (i.e., stimulus features and response features) of the same event (e.g, Colzato, Raffone, & Hommel, 2006; Hommel, 2004). On the other hand, neural synchronization can also be assumed to be a mechanism of learning (e.g., Axmacher, Mormann, Fernández, Elger, & Fell, 2006; Miltner, Braun, Arnold, Witte, & Taub, 1999). Thus, it can be speculated that short-term binding effects play a role in human learning. In fact, some theories assume that short-term associations are a first step into long-term association formation (e.g., Logan, 1988, 1990; McClelland, McNaughton, & O‘Reilly, 1995; Raffone & Wolters, 2001). In turn, short-term bindings are likely influenced by existing long-term associations.

Importantly, automatic retrieval due to short-term associations can also be triggered by an association between response irrelevant (i.e., distractor) stimuli and the response, leading to an influence on action control regarding another, currently relevant stimulus (e.g., Frings, Rothermund, & Wentura, 2007; Hommel, 2005; S. Mayr & Buchner, 2006; Rothermund, Wentura, & de Houwer, 2005). In particular, the Stimulus-Response-Retrieval theory (SRR; Rothermund et al., 2005), based on the theory of event coding (see Hommel, Müsseler, Aschersleben, & Prinz, 2001), assumes that both relevant and irrelevant stimuli become temporarily integrated in one episodic memory trace or event file together with the current response. For the duration of the event file’s existence, the repetition of any of these stimuli (i.e., also the distractor stimulus) can retrieve the entire event file, leading to a facilitation of the response that is stored in the event file and hampering different responses. This distractor-response binding effect can be analyzed in a design with two subsequent stimulus displays (i.e., a prime display and a probe display) requiring a response to each display, and orthogonally varying response and distractor repetitions. The effect is then evidenced by an interaction of response sequence and distractor sequence. In particular, the retrieval of the prime response due to a re-petition of the distractor stimulus that was presented during the prime, leads to faster probe response times if the same response is required to prime and probe. In contrast, if different responses are required to prime and probe displays, retrieval of the prime response (due to distractor repetition) leads to slower response times than presentation of different distractors. In other words, the repetition of the prime distractor as the probe distractor facilitates responding if prime and probe responses are identical, and delays responding if the probe response differs from the prime response. Distractor-response binding effects have been shown in the visual, tactile, and auditory modality, as well as across modalities, for valences, and for locations (Frings & Moeller, 2010; Frings, Moeller, & Rothermund, 2012; Frings & Rothermund, 2011; Giesen & Rothermund, 2011; S. Mayr & Buchner, 2006; S. Mayr, Buchner, & Dentale, 2009; S. Mayr, Buchner, Möller, & Hauke, 2011; Moeller & Frings, 2011; Moeller, Rothermund, & Frings, 2012).

Integrating response *irrelevant* information into event files can be understood as an adaptive default configuration of the cognitive system that allows for redundancy gains and implicit learning: On many occasions in natural settings, irrelevant features of stimuli can be assumed to be informative with regard to correct behavior because they oftentimes co-occur with relevant features within certain objects. For example, a potential predator may be identified by the shape of its body that elicits a flight response. The pattern of the predator’s fur then also becomes associated with the flight response. This association between flight and fur pattern further enhances the activation of the flight response during subsequent encounters with the predator due to a kind of redundancy gain or Garner effect (Garner & Felfoldy, 1970).

Past research on short-term stimulus-response bindings used mostly key presses that were mapped to very simple stimulus features (e.g., colors or forms). Typically, none of the varied stimulus features were strongly associated with a response from the response set (see Herwig & Waszak, 2012, for an exception). In addition, response irre-levant stimulus features (e.g., Hommel, 2005) or distractor stimuli (e.g., Giesen, Frings, & Rothermund, 2012) were either not mapped to any response or were always response incompatible. Thus, past evidence can only shed light on the role additional information plays as long as it has not been learned to be associated with a certain response. Yet, in everyday life, stimuli that are acting as distractors in certain situations may already be strongly associated to one particular action of the currently available behaviors. An interesting question is whether such a long-term association between the distractor and a particular response can prevent a short-term association between the distractor and the response given to the target (i.e., distractor-response binding). The aim of the present study is to provide first evidence to answer this question.

In fact, past findings tentatively suggest that distractor-response binding does not occur if the distractor itself is strongly associated with a response on a long-term basis (Frings & Wühr, 2007). In their second experiment, Frings and Wühr used a distractor-response binding paradigm, and presented eight different words as target and distractor stimuli. The participants’ task was to name the target word, while ignoring a distractor word. Since reading and naming are highly automatized, in this setting the distractor stimulus was always strongly associated with a particular long-term association (i.e., the particular pronunciation) that was incompatible to the response to the target word. With such strong long-term associations between distractors and response set (i.e., pronunciation of the eight stimulus words), the authors did not find evidence for short-term associations between distractors and responses (i.e., distractor-response binding). In a similar vein, Wentura and Frings (2008) analyzed evaluative priming effects with a naming task. In a first phase, participants learned to associate each picture of one set with a certain word, while pictures of a second set were not associated with specific responses. In the analysis of the main part of the experiment, priming effects in the two sets were compared, and the authors found no priming effects if the primes were bound to a naming response (that was always incompatible with the response to the target). In contrast, primes that were not associated with a specific response led to normal evaluative priming effects. Although the interpretation was somewhat different (and binding was not discussed as an explanation for the priming effects), in a nutshell, one may conclude that the integration of the response to the target and a possibly distracting stimulus hinges on whether the distractor itself elicits a learned response. Taken together, so far past results seem to suggest that distractor-response binding effects depend on the strength of association between distractor stimuli and the response-set. That is, distractor-response binding occurs if distractors are only weakly associated with the available responses. If a distractor exhibits a long-term association with one of the currently inadequate but available responses, distractor-response binding does not seem to be possible.

In the mentioned studies, strongly associated distractors were always incompatible to the currently demanded response. Thus, so far we can only speculate about the influence *compatible* long-term distractor-response association can have on distractor-response binding. Regarding a possible role short-term associations might play in human learning, it would be highly conceivable that an already existing long-term association would enhance short-term bindings between compatible responses and distractors: It seems to be adaptive to increase an already existing association between a stimulus and a certain response on each additional co-occurrence of the two. On the other hand and in line with the studies mentioned above, it would be rather maladaptive to associate a stimulus that already has a long-term association with one of the available responses with an opposing response that occurs only once together with the stimulus.

Taken together, it seems likely that the effect of distractor-response binding is influenced by the strength of long-term associations between response set and distractor stimuli. If the responses are only weakly associated with the distractors (e.g., due to the current stimulus-response mapping, see Frings & Moeller, 2012; Giesen et al., 2012), short-term bindings should be possible between both compatible and incompatible distractor-response pairings. In contrast, if the long-term associations between the distractors and the response set are strong (e.g., due to overlearning, see Frings & Wühr, 2007), short-term bindings between *incompatible* distractors and responses should be prevented, while short-term bindings between *compatible* distractors and responses should occur and possibly even be enhanced.

We used a flanker configuration of target and distractor stimuli in a prime-probe design requiring responses to both the prime and the probe display (see e.g., Frings et al., 2007) and systematically varied whether or not long-term associations existed between distractors and response-set. In addition, we controlled whether the response associated with the distractor was compatible or incompatible with the required target-response during the prime. We used simple letters as targets that were mapped via instruction to left- and right-hand responses, and we used left and right pointing arrows as distractors. To provide responses with strong long-term associations to the distractors, participants were instructed to respond via a left and a right key. Thus, each response was highly compatible to one of the possible distractors and highly incompatible to the other. In contrast, to provide responses without (or with only weak) long-term association to the distractors, participants responded via an upper and a lower key. Neither an upper nor a lower response is particularly compatible or incompatible with either distractor. Yet, since participants still responded with their left and right index fingers, we assumed that an upper response that was executed with the right index finger was still somewhat (i.e., weakly) compatible to the right, and weakly incompatible to the left pointing arrows on a long-term basis (and vice versa for the lower response that was executed with the left index finger). Note that the only difference between the conditions including strong and weak long-term associations between response-set and distractors was the location of the response keys (left/right vs. upper/lower). In both conditions, the distractor stimuli were always completely irrelevant to the task and could be ignored by the participants.

## Method

### Participants

A total of 57 students (34 women and 23 men) from the University of Trier took part in the experiment. The median age was 22 years with a range from 19 to 29 years. All participants took part for partial course credit and had normal or corrected to normal vision.

### Design

The design essentially comprised four factors. The factors Response Sequence (repetition vs. alternation) and Distractor Sequence (repetition vs. alternation) were varied within participants, whereas the factor Strength of Long-Term Distractor/Response-Set Association (strong association vs. weak association) was varied between participants. In addition, distractors were compatible to the response in half of the prime displays and incompatible in the other half.

### Materials

The experiment was conducted using the E-prime software (E-prime 2.0). Instructions and all stimuli were shown in white on black background on a standard CRT screen. The target stimuli were the letters *D*, *F*, *J*, and *K*; and the distractors consisted of the arrow signs “<” and “>,” that were presented in triplets (e.g., “>>>”). The letters were 1.1 cm wide and 1.0 cm high. Each distractor stimulus triplet was 3.2 cm wide and 1.0 cm high. The combination of one target letter and two distractor triplets extended 7.6 cm horizontally and 1.0 cm vertically. A constant viewing distance of 50 cm was provided by asking participants to place their heads on a chin rest.

### Procedure

**Figure 1.**
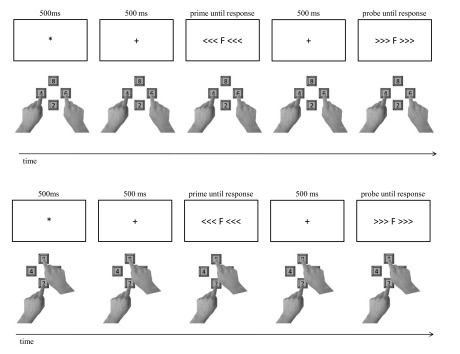
Sequence of events in one trial in the condition with strong distractor/response-set association (upper panel) and the condition with weak distractor/response-set association (lower panel). In both conditions, participants responded via the number pad to the identity of the letter (left index finger for *D* and *F*; right index finger for *J* and *K*) and ignored the flanking arrows. White is depicted in black and black is depicted in white. Stimuli and keys are not drawn to scale.

Participants were tested individually in soundproof chambers. Instructions were given on the screen and summarized by the experimenter. All participants responded to the letters *D* and *F* by pressing a key with their left index finger and to the letters *J* and *K* by pressing a key with their right index finger. Participants responded via the number pad of a standard keyboard. The number keys “8,” “5,” and “2” were centrally aligned with the computer screen. The only difference between the two conditions was the location of participants’ index fingers. In the condition with strong long-term distractor/response-set association, participants were instructed to place their left index finger on the key “4” (the left key), and their right index finger on the key “6” (the right key), whereas participants in the condition with weak long-term distractor/response-set association were instructed to place their left index finger on the key “2” (the lower key) and their right index finger on the key “8” (the upper key). In the strong association condition, the instructions always referred to the left and right keys, while in the weak association condition it always referred to the keys “8” and ”2”. Participants’ task was always to identify the target letter by pressing the corresponding key and to ignore the flanking arrows. Participants always saw one target letter that was flanked by three arrows on both sides that all pointed in the same direction (e.g., “<<<*F*<<<”). A single trial (prime/probe sequence) consisted of the following sequence of events (cf. Figure 1): At the beginning of each trial an asterisk was presented in the center of the screen to inform the participant that the trial started. After 500 ms the asterisk was exchanged for a white plus sign that served as a fixation mark. After another 500 ms the prime display, consisting of one letter flanked by two arrow triplets, was presented until participants’ response. After the prime response, again the fixation mark was shown for 500 ms, followed by the probe display. Similar to the prime display, the probe also consisted of a letter that was flanked by two arrow triplets and stayed on the screen until participants responded.

In response repetition (RR) trials, the same response was required to the prime letter and to the probe letter. For example, if the prime target was *D*, the probe target could be *F* (requiring a left-left response in the condition with strong long-term distractor/response-set association). In response alternation (RA) trials, the response required during the probe differed from that required during the prime. For example, if the prime target was *D*, the probe target could be *J* (requiring a left-right response in the condition with strong long-term distractor/response-set association). Orthogonally to the response sequence, the distractor sequence was varied. In distractor repetition (DR) trials, the arrows (i.e., distractors) pointed in the same direction on the prime and on the probe. In distractor alternation (DA) trials, the arrows on the probe pointed in the opposite direction of those on the prime. In turn, four different conditions were conducted both for weak and for strong long-term distractor/response-set associations: In RRDR trials, the prime response and the prime distractor were repeated on the probe. In RRDA trials, the prime response was repeated as the probe response while the distractor alternated from prime to probe. In RADR trials, the probe response differed from the prime response while the prime distractor was repeated as the probe distractor. Finally, in RADA trials, neither response nor distractor was repeated from the prime to the probe.

Each participant worked through an experimental block consisting of 320 prime-probe sequences. The four trial types (RRDR, RRDA, RADR, and RADA) were realized in 80 trials each. One of the letters *D*, *F*, *J*, and *K* was randomly assigned to the probe target. A second of the letters was randomly assigned to the prime target with the constraint that the required response was the same during prime and probe in the case of RR trials and different in the case of RA trials. In half of the RR trials, the same letter was presented during prime and probe, and in the other half, different letters that were mapped to the same response were presented during the prime and the probe. Orthogonally to these conditions, the arrow-distractors during the prime pointed to the left side in half of the trials and to the right side in the other half. According to the distractor sequence on the current trial, the same or the opposite arrow was assigned to the probe distractor. During the experimental block, participants could take a short break every 64 trials. Before the experimental block started, participants worked through a practice block of 32 prime-probe sequences, during which participants received feedback after each response.

## Results

If not mentioned differently, the significance level was set to *p* = .05 (two-tailed) in all analyses. In the analyses of probe reaction times (RTs), only trials with correct answers to the prime and the probe were considered. RTs that were more than 1.5 interquartile ranges above the third quartile of the RT distribution of each participant (Tukey, 1977), and those that were shorter than 200 ms were excluded from the analysis. Due to these constraints, 13.7% of all trials were discarded (probe error rate was 4.3%, prime error rate was 4.7%). Mean RTs and error rates for probe displays are depicted in Table 1. In the analyses of prime RTs, only trials adhering to analogue criteria as were used for the probe RTs were considered. Due to these constraints, 10.3% of all prime trials were discarded. Prime RTs for weak long-term distractor/response-set association (603 ms) did not differ from prime RTs for strong long-term distractor/response-set association (585 ms), *t*(55) = 1.07, *p* = .288.

**Table 1.** Mean Reaction Times (in ms) and Mean Error Rates (in percentage) as a Function of Response and Distractor Sequence, Strength of Distractor/Response-Set Association on the Prime, and Compatibility of Distractor and Response on the Prime

	Strong long-term distractor/response-set association		Weak long-term distractor/response-set association	
	Response repetition	Response alternation	Response repetition	Response alternation
Compatible				
Distractor alternation	580 (4.1)	603 (2.1)	584 (2.8)	628 (2.5)
Distractor repetition	549 (2.0)	616 (3.6)	584 (2.5)	632 (3.7)
Incompatible				
Distractor alternation	554 (2.5)	616 (2.2)	578 (2.2)	624 (2.1)
Distractor repetition	561 (2.0)	607 (2.5)	569 (2.4)	630 (3.3)

### Distractor-response compatibility manipulation check

To determine whether our manipulation of distractor-response compatibility was successful, we conducted a mixed models MANOVA on prime RTs with the factors Long-Term Distractor/Response-Set Association Strength (between participants) and Prime Distractor-Response Compatibility (within participants). The main effect of compatibility was significant, *F*(1, 55) = 10.27, *p* = .002, η_p_^2^ = .16. The interaction of Long-Term Distractor/Response-Set Association Strength with Distractor-Response Compatibility did not reach significance, *F*(1, 55) = 1.39, *p* = .244, η_p_^2^ = .03. Yet, separate analyses revealed that the compatibility effect was only significant if the long-term association between distractor and response was strong, *t*(28) = 2.91, *p* = .007, but not if the association was weak, *t*(27) = 1.55, *p* = .133. For the prime RTs and error rates, see Table 2.

**Table 2.** Prime Reaction Times (in ms) and Percent Errors (in parentheses) as a Function of Distractor-Response Compatibility and Distractor/Response-Set Association Strength

	Strong long-term distractor/response-set association	Weak long-term distractor/response-set association
Compatible	569 (3.4)	580 (4.9)
Incompatible	578 (5.1)	585 (5.5)

### Analysis of probe reaction times

In RR trials, it was orthogonally varied whether the target did or did not repeat from prime to probe. Since it has been shown that distractor-target bindings can influence RTs in addition to distractor-response bindings (Giesen & Rothermund, 2014), whether or not the target was repeated might have had a modulating effect in the result pattern. Hence, to control for a possible confound of target and response repetitions (cf. Frings et al., 2007; Giesen et al., 2012; Wendt & Luna-Rodriguez, 2009), we conducted all analyses also with the factor Target Repetition/Change. The factor did not have a modulating influence on distractor-response binding, *F*(1, 55) = 0.41, *p* = .526, η_p_^2^ = .01; distractor/response-set association strength, *F*(1, 55) = 1.17, *p* = .285, η_p_^2^ = .02; the interaction of Distractor-Response Binding and Distractor/Response-Set Association Strength, *F*(1, 55) = 0.16, *p* = .695, η_p_^2^ = .003; or on the interaction of Distractor-Response Binding, Associations Strength, and Distractor-Response Compatibility, *F*(1, 55) = 1.65, *p* = .205, η_p_^2^ = .03. For the sake of clarity, we therefore averaged over target repetitions and target alternations.

**Figure 2.**
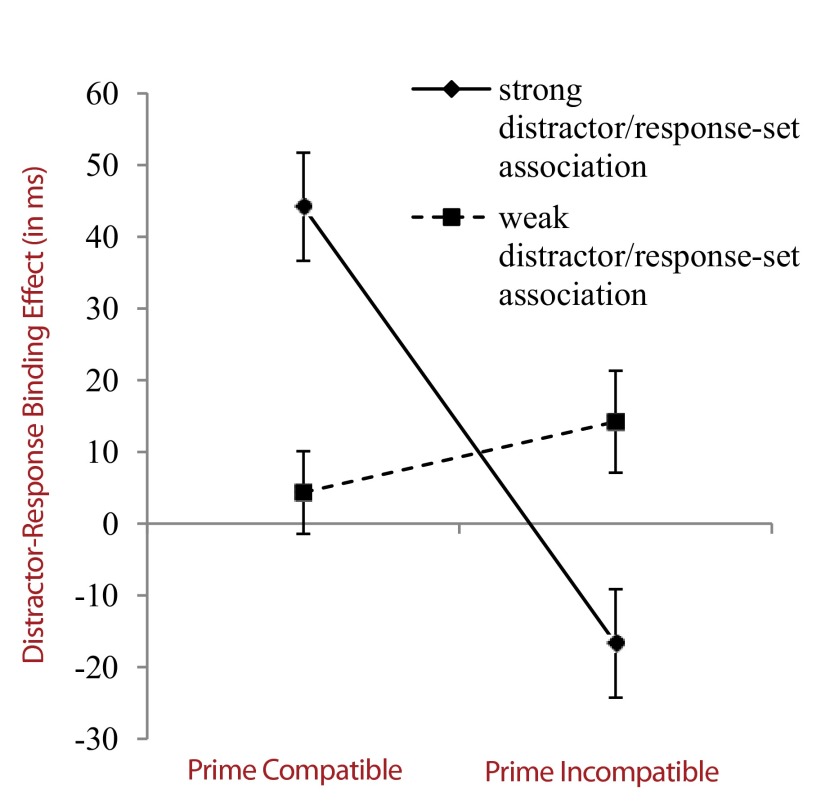
Distractor-response binding effect (in milliseconds) as a function of distractor/response-set association and compatibility of the prime distractor to the prime response. Distractor-response binding effects are computed as the difference between the distractor repetition effects in response repetition and response alternation trials.

A 2 × 2 × 2 × 2 MANOVA on probe RTs with the within-subject factors Response Sequence (repetition vs. alternation), Distractor Sequence (repetition vs. alternation), and Distractor-Response Compatibility During the Prime (compatible vs. incompatible), and the between-subjects factor Strength of Long-Term Distractor/Response-Set Association (strong association vs. weak association) was conducted to analyze whether prime compatibility differentially influenced distractor-response binding in the strong and weak distractor/response-set association conditions. The main effects of response sequence, *F*(1, 55) = 235.32, *p* < .001, η_p_^2^ = .81; and prime compatibility, *F*(1, 55) = 10.65, *p* = .002, η_p_^2^ = .16; were significant. Responses were faster in RR (570 ms) than in RA trials (619 ms), and responses were faster after incompatible (592 ms) than after compatible (596 ms)primes. The effect of distractor-response binding was significant, *F*(1, 55) = 14.44, p < .001, η_p_^2^ = .21; as well as the interaction of the Distractor-Response Binding Effect with Distractor-Response Compatibility During the Prime, *F*(1, 55) = 10.55, *p* = .002, η_p_^2^ = .16, indicating a larger effect of distractor response binding after distractor-response compatible primes than after distractor-response incompati-ble primes. Most importantly, the four-way interaction was significant as well, *F*(1, 55) = 19.92, *p* < .001, η_p_^2^ = .27, indicating that the influence distractor-response compatibility during the prime had on the distractor-response binding effect was significantly different for weak and strong long-term distractor/response-set associations (cf. Figure 2). For the sake of completeness, the interaction of Response Sequence with Prime Compatibility was significant as well, *F*(1, 55) = 5.99, *p* = .018, η_p_^2^ = .10.

The same MANOVA on error rates revealed a similar pattern. The effect of distractor-response binding was significant, *F*(1, 55) = 22.33, *p* < .001, η_p_^2^ = .29, as well as the interaction of Distractor-Response Compatibility During the Prime with the Effect of Distractor-Response Binding, *F*(1, 55) = 6.21, *p* = .016, η_p_^2^ = .10. The four-way interaction of Distractor-Response Compatibility During the Prime × Strength of Long-Term Distractor/Response-Set Association × Distractor-Response Binding Effect did not reach significance, *F*(1, 55) = 2.50, *p* = .119, η_p_^2^ = .04.

To further analyze the interaction of Distractor/Response-Set Association Strength, Prime Compatibility, and the Distractor-Response Binding Effect, separate analyses for weak and strong long-term distractor/response-set association were conducted.

### Strong long-term distractor/response-set association

**Figure 3.**
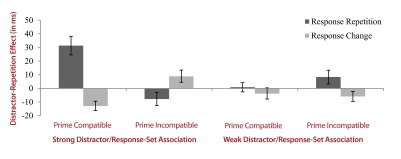
Distractor repetition effects in milliseconds (probe reaction times in distractor alternation minus distractor repetition trials) as a function of response sequence, distractor-response compatibility on the prime, and distractor-/response-set association. Error bars depict the standard errors of the means.

In a within-subject 2 (Response Sequence: repetition vs. alternation) × 2 (Distractor Sequence: repetition vs. alternation) × 2 (Distractor-Response Compatibility During the Prime: compatible vs. incompatible) MANOVA on probe RTs, the interaction of Response Sequence and Distractor Sequence was significant, *F*(1, 28) = 8.36, *p* = .007, η_p_^2^ =.23, indicating an effect of distractor-response binding. Importantly, the distractor-response binding effect was modulated by distractor-response compatibility during the prime, indicated by a significant interaction of the Distractor-Response Binding Effect with Distractor-Response Compatibility During the Prime, *F*(1, 28) = 27.03, *p* < .001, η_p_^2^ = .49. Separate analyses showed that the effect of distractor-response binding was significant if the prime distractor was compatible to the prime response, *F*(1, 28) = 34.40, *p* < .001, η_p_^2^ = .55, but not if prime distractor and response were incompatible. In fact, if prime distractors and responses were incompatible, the repetition of the distractor seemed to facilitate the response that had not been shown on the prime, *F*(1, 28) = 4.91, *p* = .035, η_p_^2^ = .15 (i.e., a reversed distractor-response binding effect1; for the distractor-repetition effects, see Figure 3, left panel).

The same MANOVA on the error rates revealed similar results. The distractor-response binding effect was significant, *F*(1, 28) = 16.99, *p* < .001, η_p_^2^ = .38, as well as the interaction of the Distractor-Response Binding Effect with Distractor-Response Compatibility During the Prime, *F*(1, 28) = 8.02, *p* = .008, η_p_^2^ = .22.

### Weak long-term distractor/response-set association

In a within subjects 2 (Response Sequence: repetition vs. alternation) × 2 (Distractor Sequence: repetition vs. alternation) × 2 (Distractor-Response Compatibility During the Prime: compatible vs. incompatible) MANOVA on probe RTs, the effect of distractor-response binding was significant, *F*(1, 27) = 6.24, *p* = .019, η_p_^2^ = .19. Importantly, the interaction of the Distractor-Response Binding Effect with Distractor-Response Compatibility During the Prime was not significant, *F*(1, 27)= 0.83,*p* = .371, η_p_^2^ = .03. That is, for weak long-term distractor/response-set association, the compatibility of distractor and response during the prime did not modulate the effect of distractor-response binding (for the distractor repetition effects, see Figure 3, right panel).

The same MANOVA on the error rates showed an identical pattern. The effect of distractor-response binding was significant, *F*(1, 27) = 6.37, *p* = .018, η_p_^2^ = .19, while the interaction of this effect with Distractor-Response Compatibility During the Prime was not, *F*(1, 27) = 0.43, *p* = .52, η_p_^2^ = .02.

### Sequential compatibility effects

**Figure 4.**
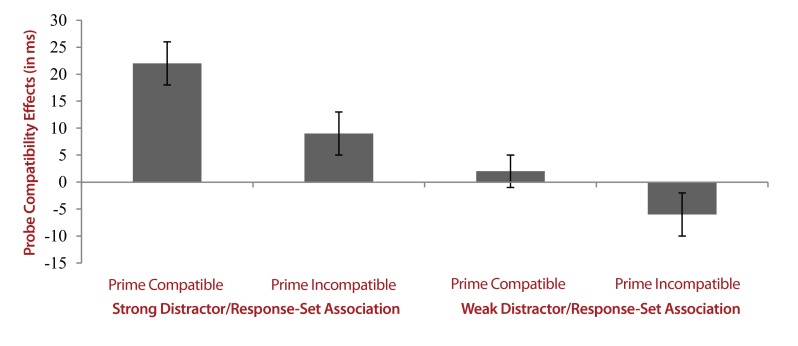
Probe compatibility effects in milliseconds (reaction times to incompatible probes minus reaction times to compatible probes) as a function of distractor-response compatibility on the prime and distractor-/response-set association strength. Error bars depict the standard errors of the means.

With response-compatible and response-incompatible distractors we have to consider the role sequential compatibility effects played in the experiment. In a sequential design, as used in the present study, compatibility effects on the probes would be smaller after incompatiblethan after compatible primes (Gratton effect; see Gratton, Coles, & Donchin, 1992; for the relations between trial types of the Gratton- and distractor-response binding effect, see Table 3). With the present design the Gratton effect would enhance the pattern of distractor-response binding. Due to the nature of the distractor stimuli and the responses (a distractor was always compatible to one of the responses and incompatible to the other), after a response compatible prime distractor, probe distractors were compatible on RRDR and RADA trials but incompatible on RADR and RRDA trials (and vice versa for incompatible prime distractors). Thus sequential compatibility effects would have led to response facilitation in RRDR and RADA trials as compared to RADR and RRDA trials, enhancing the distractor-response binding pattern. Thus, we analyzed whether the data pattern can also be explained by a modulation of the Gratton effect by the association strength between distractors and response-set. We conducted a mixed models MANOVA on probe RTs with the within subjects factors Prime Compatibility (compatible vs. incompatible) and Probe Compatibility (compatible vs. incompatible), and the between subjects factor Strength of Long-Term Distractor/Response-Set Association (strong association vs. weak association). (For the mean RTs and the error rates, see Table 4.) The main effects of prime compatibility, *F*(1, 55) = 10.02, *p* = .003, η_p_^2^ = .15, and probe compatibility, *F*(1, 55) = 10.71, *p* = .002, η_p_^2^ = .16, were significant. Participants responded faster if prime distractors were incompatible (592 ms) than if prime distractors were compatible (597 ms),and they responded faster if probe distractors were compatible (591 ms)than if probe distractors were incompatible (598 ms). The interaction of Prime Compatibility and Probe Compatibility was significant as well, *F*(1, 55) = 12.80, *p* = .001, η_p_^2^ = .20, indicating larger compatibility effects after compatible than after incompatible primes. In addition, the interaction of Probe Compatibility and Distractor/Response-Set Association Strength was also significant, *F*(1, 55) = 18.37, *p* < .001, η_p_^2^ = .25, showing larger probe compatibility effects with the strong association as compared to the weak association (cf. Figure 4). Importantly, the sequential compatibility effect was not modulated by distractor/response-set association strength, *F*(1, 55) = 0.63, *p* = .430, η_p_^2^ = .01. Sequential compatibility effects were significant both for strong distractor/response-set associations, *F*(1, 28) = 8.17, *p* = .008, η_p_^2^ = .23, and also for weak distractor/response-set associations, *F*(1, 27) = 4.75, *p* = .038, η_p_^2^ = .15. The same MANOVA for error rates revealed results along the same lines. The main effects of prime compatibility, *F*(1, 55) = 6.35, *p* = .015, η_p_^2^ = .10, and probe compatibility, *F*(1, 55) = 6.21, *p* = .016, η_p_^2^ = .10, were significant. The sequential compatibility effect, *F*(1, 55) = 22.33, *p* < .01, η_p_^2^ = .29, was also significant but was not modulated by distractor/response-set association strength, *F*(1, 55) = 1.62, *p* = .208, η_p_^2^ = .03.

**Table 3.** Relation Between Trial Types of the Gratton- and Distractor-Response Binding Effect

Distractor-response binding			Sequential compatibility		
RRDR	includes	c-c/i-i	c-c	includes	RRDR/RADA
RRDA	includes	c-i/i-c	c-i	includes	RRDA/RADR
RADR	includes	c-i/i-c	i-c	includes	RRDA/RADR
RADA	includes	c-c/i-i	i-i	includes	RRDR/RADA

*Note*. c-c = prime compatible – probe compatible, c-i = prime compatible – probe incompatible, i-c = prime incompatible – probe compatible, i-i = prime incompatible – probe incompatible. DA = distractor alternation, DR = diatractor repetition, RA = response alternation, RR = response repetition.

**Table 4.** Mean Reaction Times (in ms) and Mean Error Rates (in percentage) as a Function of Prime Compatibility, Probe Compatibility, and Strength of Distractor/Response-Set Association

	Strong long-term distractor/response-set association		Weak long-term distractor/response-set association	
	Probe compatible	Probe incompatible	Probe compatible	Probe incompatible
Prime compatible	576 (4.1)	598 (7.7)	606 (5.0)	608 (6.5)
Prime incompatible	580 (5.0)	589 (4.2)	604 (5.5)	597 (4.5)

## Discussion

The purpose of the present experiment was to systematically investigate the influence of long-term associations between responses and distractor stimuli on distractor-response binding effects (i.e., short-term distractor-response associations). We compared the effect of distractor-response binding in a condition using a response-set that was strongly associated with the distractor stimuli on a long-term basis, with the same effect in a condition with weak long-term associations between response-set and distractors. Participants always responded to target letters that were mapped via instruction either to a left and a right or to a lower and an upper response (executed with the left and right hand, respectively). Distractors were arrows pointing left or right but were completely irrelevant to the task. Further, in 50% of the prime and probe displays, the distractors were compatible and in the other 50% they were incompatible with the response hand. That is, in both conditions participants could not rely on the direction of the distractors to improve their performance.

## Interpretation in terms of stimulus-response binding

We found an influence of distractor-response compatibility on the effect of distractor-response binding only in the strong association condition but not in the weak association condition. If the response-set (i.e., left and right button presses) was strongly associated with the distractor set (i.e., left and right pointing arrows) on a long-term basis, response-compatible distractors were integrated with the prime responses and a repetition of such a distractor subsequently retrieved this response. However, if the strong long-term response association of the prime distractor was incompatible with the required prime response, binding of distractor and response did not occur, and a repetition of the distractor did not retrieve that response. In contrast, compatibility of prime distractor and response did not modulate distractor-response binding if distractors and responses were only weakly associated on a long-term basis. Moreover, the result patterns for strong and weak long-term association conditions differed significantly.

Notably, the distractors in the weak-association condition had the same long-term association with a certain (i.e., left or right) response as the distractors in the strong-association condition. The important difference between the two conditions was whether the associated responses were part of the current response-set. This indicates that stimuli that are strongly associated with currently available responses have a different influence on action control than stimuli that are not (long-term) associated with a response of the currently available response set. In turn, the potential link to learning mechanisms becomes more plausible: New short-term associations between stimuli and responses can be formed relatively easily, while existing long-term associations prevent opposing short-term bindings (that might weaken the learned association) and enhance additional short-term binding of the long-term association (possibly further strengthening the long-term association). One mechanism ensuring formation of mainly performance enhancing short-term associations may rely on conflict perception (see Egner, 2008; Wiswede, Rothermund, & Frings, 2013). Strong conflict due to long-term associations between distractors and responses might prevent integration of these. Such conflict occurred only for incompatible primes of our strong distractor/response-set association group in the present experiment. Note that strong conflict is also assumed to enhance focusing cognitive resources on the subsequent display (see Botvinick, Braver, Barch, Carter, & Cohen, 2001; Wendt, Kluwe, & Vietze, 2008). That is, following strong conflict, more cognitive control impeding distractor encoding might prevent possible effects of distractor-based response retrieval.

Yet, still more research is necessary to better understand the relationship between binding and learning. For example, Herwig and Waszak (2012) report evidence regarding action-effect bindings in a learning task. Whether participants were prompted to execute a certain response or were allowed to decide which response they would show, influenced learning of action-effect associations but had no modulating influence on short-term action-effect bindings (cf. Experiment 3).Even though these findings indicate that learning and short-term bindings may be influenced by different factors, the study does not allow conclusions whether the learned associations do or do not have an influence on short-term bindings. Regarding the present results, one might speculate that long-term associations would also modulate the effect of action-effect bindings.

In addition, our findings are in line with the assumption that human actions are influenced by stimulus characteristics that have been learned to be relevant for the planned action (Wykowska, Hommel, & Schubö, 2011; see also Hommel et al., 2001; Wykowska, Schubö, & Hommel, 2009). That is, if an action-relevant characteristic is encountered, its processing is prioritized. Further, the dimensional overlap model (Kornblum, 1992, 1994; Kornblum et al., 1990) specifically proposes that also an overlap between dimensions of *irrelevant* stimuli and responses affects performance (see also Lu & Proctor, 2001). In the present study, the direction of the distractor arrows (left/right) was action-relevant only in the strong long-term association condition (left/right responses), but not in the weak association condition (upper/lower responses). Thus, we can assume that the processing of distractors was only prioritized in the former but not in the latter condition. This might have enhanced the effect of distractor-response binding with strong long-term distractor/response-set association. Yet, prioritized distractor processing alone cannot account for the different effects of prime distractor-response compatibility and incompatibility in the condition with a strong long-term distractor/response-set association. Prioritized distractor processing would predict different effects for strong and weak long-term distractor/response-set associations but the same effect for response compatible and incompatible prime distractors. However, we found that the effect of distractor-response binding was not generally modulated by the strength of long-term distractor/response-set association, *F*(1, 55) < 1.0, *p* > .47, η_p_^2^ = .009. In addition, the effect of distractor-response binding in the strong association condition did not occur if prime distractor and response were incompatible. Taken together, our data only partially fit to an explanation in terms if prioritized distractor processing.

### Interpretation in terms of the Gratton effect

Interpreted differently, our results evidence sequential modulations of the compatibility effect (i.e., the Gratton effect; Gratton et al., 1992) and must also be discussed through the lens of the Gratton effect. With both compatible and incompatible distractors, the observed pattern might also be explained via sequential adjustments of compatibility effects: Performance was better in trials that included a repetition of the compatibility type (RRDR and RADA trials) than in trials that included compatibility type alternation (RADR and RRDA trials; for effects of compatibility type repetition in the weak and strong association conditions, see Figure 4). More specifically, distractor-response compatibility effects on the probe were smaller after incompatible primes than after compatible primes. One possibility to account for such effects is to assume an adaptation of the cognitive system to the amount of conflict perceived during the response on a previous trial (Gratton et al., 1992; see also Botvinick et al., 2001; another possibility is to explain the Gratton effect due to partial matches between consecutive trials, see e.g., Hommel, Proctor, & Vu, 2004; U. Mayr, Awh, & Laurey, 2003; Notebaert, Soetens, & Melis, 2001). After experiencingconflict in an incompatible prime, more cognitive control on the following display ensures less influence due to distractor stimuli. The Gratton effect is typically tested in a design using the same stimulus set as targets and distractors (e.g., Botvinick, Nystrom, Fissell, Carter, & Cohen, 1999; Gratton et al., 1992; Verbruggen, Notebaert, Liefooghe, & Vandierendonck, 2006; Wendt, Kluwe, & Peters, 2006). Consequently, compatibility effects are driven by distractors that are mapped to different or the same response as indicated by the target.

In our experiment, the distractor stimuli were never presented as targets and were not mapped to any of the responses. Instead, compatibility effects were due to the fact that participants responded with their left and right hand and the response irrelevant distractors were left and right pointing arrows. Nevertheless, we found a pattern that can be interpreted as a Gratton effect. That is, even though distractors were not mapped to the response set, and participants never responded to these stimuli, long-term associations between distractor stimuli and responses led to conflict and in turn to conflict adaptation. This effect was not modulated by association strength of distractors and response-set. A closer look at the data reveals a difference between the patterns after compatible than after incompatible primes only if distractor/response-set association was strong but not if it was weak. This difference is not predicted by an account of the Gratton effect but can be accounted for if distractor-response binding is assumed to be prevented between highly incompatible stimuli and responses. Thus, we can assume that the effect of distractor-response binding at least in part contributed to the result pattern. Yet, to clearly differentiate Gratton and binding effects, further research is necessary.

Since the Gratton effect was not modulated by distractor/response-set association strength, the pattern may suggest that the conflict leading to Gratton effects was caused by the long-term association distractor stimuli had with the *effectors* rather than the *labels* of the responses. Right arrows were associated with right hand responses, while left arrows were associated with left hand responses both in weak and in strong distractor/response-set association conditions. In fact, effectors have been shown to play an important role for sequential modulation of compatibility effects. Braem, Verguts, and Notebeart (2011) found Gratton effects across task switches only if the effector set repeated between tasks (hands-hands) but not if the effector switched (hands-feet). Yet, in feature-response bindings, not the effector but rather the response code is integrated in an event file (Stoet & Hommel, 1999). The modulating effect of association strength in trials with incompatible primes is in line with this finding: Integration was prevented for strongly incompatible *response codes* and responses.

The current result pattern is also similar to the results obtained by Schlaghecken and Martini (2012), who investigated behavioral adjustments after conflict and non-conflict trials. As in the present experiment, the authors orthogonally varied response and distractor-feature relation but interpreted the latter as trial type relation. Thus, they did not include into their reasoning whether or not the distractor feature (i.e., the cue, the prime, or the irrelevant target position) alternated from trial *n* - 1 to trial *n*. Consequently, they did also not discuss an effect of bindings between these features and responses. Yet, the same pattern as in the present experiment can be seen in their Figure 2: The pattern of distractor-response binding was more pronounced if trial *n* - 1 was compatible than if it was incompatible. In contrast to the study by Schlaghecken and Martini (2012), the general effect of conflict repetition benefit in our experiment was larger for incompatible-incompatible sequences than for compatible-compatible sequences, *F*(1, 56) = 6.11, *p* = .017, η_p_^2^ = .10. An obvious difference between the studies is the participants’ task: The authors of the cited study analyzed cuing-, priming-, and Simon-tasks, while we used a flanker task. It is possible that binding mechanisms and/or context adaptation mechanisms work differently for additional but simultaneously appearing stimuli than for response irrelevant target features or stimuli that precede the target. Yet, with the present results we cannot decide whether the differences in the conflict repetition benefits can be accounted for by the task difference or by other differences between the experiments. Thus, we have to leave this question to be answered by future research.

### Relation to previous findings

Finally, we will also discuss our data with respect to some constraints and possible conflicts with prior studies. First, it should be mentioned that in addition to the reported effects, distractor-response compatibility during the probe may have influenced the present results. A distractor stimulus was always compatible to one of the responses and incompatible to the other. Thus, after a response compatible prime distractor, probe distractors were compatible on RRDR and RADA trials but incompatible on RADR and RRDA trials (and vice versa for incompatible prime distractors). This constraint led to an overestimation of the effect of distractor-response binding if prime distractor and response were compatible but to an underestimation of the distractor-response binding effect if prime distractor and response were incompatible. Note, however, that this effect cannot explain the general effects of distractor-response binding in the strong and weak distractor-response set association conditions (the pattern caused by probe compatibility must have cancelled out between trials with compatible and incompatible primes). Given the non-significant difference between compatibility effects in the prime RTs of the strong and the weak association condition, the different effects of prime compatibility on the distractor-response binding effect in the strong versus the weak association conditions are at best unlikely to be due to differences in compatibility effects in the weak and strong association conditions (however, given the confound with compatibility a final conclusion on whether stimulus-response binding is prevented by long-term associations cannot be drawn).

Interestingly, we found that probe responses following incompatible primes were faster than following compatible primes. In contrast, it has been shown that conflict can lead to slower responses on subsequent trials (Verguts, Notebaert, Kunde, & Wühr, 2011). In the present experiment, the effect seemed to be partly due to relatively fast responses in RRDA and RADR trials after incompatible primes in the strong association condition. On the one hand, in RRDA and RADR trials, incompatible primes are followed by compatible probes. In addition, in the strong association condition, incompatible prime distractors were not integrated with a response that would have slowed down probe responses in RRDA and RADR trials. That is, the post-conflict advantage was likely due to a combination of probe compatibility effects and the reported modulation of distractor-response binding.

At first sight, the present findings are inconsistent with various studies suggesting that nearly any irrelevant stimulus feature can be integrated with and later on retrieve a response (e.g., Hommel, 2005; Notebaert & Soetens, 2003; Zmigrod & Hommel, 2011). For example, Notebaert and Soetens (2003) report a result pattern that indicates response retrieval due to feature repetition even if the feature has to be ignored in order to carry out the correct response. Hommel (2005) presented evidence that hardly any attention to a stimulus feature is necessary for it to be integrated into the same event file with the response. Yet, these findings do not contradict the present results as none of these studies used responses that were strongly associated with the stimuli. Stimulus-response mappings were only established during the instructions of the experiments (regarding the response relevant features) or not at all (regarding additional features). Thus, modulating effects of long-term stimulus-response associations could not be analyzed.

More relevant in this regard is the study of Colzato and colleagues (2006). They investigated the influence of long-term bindings between stimulus features on short-term feature bindings and found no modulating effect of long-term associations on short-term bindings. In turn, they concluded that long-term feature associations have no direct influence on short-term feature bindings. Of course, the same logic as above can be applied here, namely, that in the experiments of Colzato et al. stimuli were not long-term associated with responses.

Yet, a closer look at the data of Colzato et al. (2006) suggests a different interpretation. In their Experiment 3 (using the highly overlearned feature combinations: red-strawberry and yellow-banana), the authors found no difference between partial repetition costs for familiar and unfamiliar feature combinations on the *second* of the two responses. To decide whether binding is influenced by long-term associations, it would be interesting to compare effects of partial repetition as a function of familiarity of feature conjunctions in the first of the two responses. In fact, from a mere comparison of the means presented in their Figure 5, it looks as though this interaction of S1 familiarity with partial repetition costs might be significant, with partial repetition costs for familiar (e.g., yellow-banana), but not for unfamiliar (e.g., yellow-strawberry) feature combinations. However, with the additional effect of compatibility/incompatibility in the second display, these RTs are difficult to interpret. Yet, it is possible that short-term feature bindings are modulated by long-term associations between stimulus features as well. However, certainly more research is required to validate this speculation or more specifically analyze why short-term distractor-response but not feature bindings are influenced by long-term associations.

### Conclusion

Taken together, several past studies evidence that even task irrelevant, ignored stimuli can be integrated with and later retrieve a response and thereby influence our behavior. Since people encounter most objects not only once but regularly in everyday life, it can be assumed that most stimuli have been associated with other stimuli and/or responses on a long-term basis. Here we investigated the influence established long-term stimulus-response associations have on distractor-response binding, that is, a short-term mechanism of action control. The present results indicate that the influence of ignored stimuli depends on our past experience with these stimuli. If a distractor is strongly associated with a currently available response on a long-term basis, this stimulus is unlikely to become part of a short-term association with a different response. Thus, our cognitive system uses only the irrelevant information from our environment which is free of long-term stimulus-response associations to improve behavior. Thereby automatic stimulus-response retrieval of established behavioral routines is not endangered by distractor-response binding.

## Author Note

The research reported in this article was supported by a grant of the Deutsche Forschungsgemeinschaft to Christian Frings (FR 2133/1-2).
